# JSQE: Joint Surveillance Quality and Energy Conservation for Barrier Coverage in WSNs

**DOI:** 10.3390/s22114120

**Published:** 2022-05-29

**Authors:** Xuemei Shao, Chih-Yung Chang, Shenghui Zhao, Chin-Hwa Kuo, Diptendu Sinha Roy, Xinzhe Pi, Shin-Jer Yang

**Affiliations:** 1 School of Computer and Information Engineering, Chuzhou University, Chuzhou 239000, China; sxm@chzu.edu.cn (X.S.); zsh@chzu.edu.cn (S.Z.); 2 Department of Computer Science and Information Engineering, Tamkang University, New Taipei City 25137, Taiwan; cychang@mail.tku.edu.tw; 3 Department of Computer Science and Engineering, National Institute of Technology, Shillong 793003, India; diptendu.sr@nitm.ac.in; 4 School of Systems Information Science, Future University Hakodate, Hakodate 041-8655, Japan; pixinzhe@gmail.com; 5 Department of Computer Science and Information Management, Soochow University, Taipei 100006, Taiwan; sjyang@csim.scu.edu.tw

**Keywords:** wireless sensor networks, barrier coverage, ESM, surveillance quality

## Abstract

Barrier coverage is a fundamental issue in wireless sensor networks (WSNs). Most existing works have developed centralized algorithms and applied the Boolean Sensing Model (BSM). However, the critical characteristics of sensors and environmental conditions have been neglected, which leads to the problem that the developed mechanisms are not practical, and their performance shows a large difference in real applications. On the other hand, the centralized algorithms also lack scalability and flexibility when the topologies of WSNs are dynamically changed. Based on the Elfes Sensing Model (ESM), this paper proposes a distributed Joint Surveillance Quality and Energy Conservation mechanism (JSQE), which aims to satisfy the requirements of the desired surveillance quality and minimize the number of working sensors. The proposed JSQE first evaluates the sensing probability of each sensor and identifies the location of the weakest surveillance quality. Then, the JSQE further schedules the sensor with the maximum contribution to the bottleneck location to improve the overall surveillance quality. Extensive experiment results show that our proposed JSQE outperforms the existing studies in terms of surveillance quality, the number of working sensors, and the efficiency and fairness of surveillance quality. In particular, the JSQE improves the surveillance quality by 15% and reduces the number of awake sensors by 22% compared with the relevant TOBA.

## 1. Introduction

Wireless sensor networks (WSNs) consist of many sensor nodes, which are capable of sensing and processing data in a monitoring region. Sensor nodes can communicate with one another through data exchange in a wireless manner. WSNs have been widely applied in many applications, such as health monitoring, agriculture, and environmental monitoring [1,2,3,4].

Network coverage is known as one of the most important issues in wireless sensor networks. It can be divided into three categories [5,6,7,8,9]: target coverage, area coverage, and barrier coverage. Target coverage aims to detect some interesting targets using working sensors, while the area coverage concerns the monitoring coverage over a predefined region. In contrast to the target and area coverages, barrier coverage investigates intruder detections when crossing boundaries or interesting curves. The main purpose of barrier coverage is to form a defense barrier by organizing some working sensors to detect intruders crossing international boundaries, or detect the spread of forest fires around forests. It also has been widely applied to protect critical areas and important resources, including military bases and the alarm boundaries for visitors in certain zones.

In recent years, the surveillance quality for barrier coverage issues in WSNs has received a large amount of attention. Many studies have presented algorithms to form a defense barrier with k-barrier coverage [10] or strong barrier [11] capabilities, which guarantees monitoring quality so that every intrusion can be detected by sensors participating in the barrier. The sensing models applied in these studies can be mainly divided into two categories, namely the Boolean Sensing Model (BSM) [12,13,14,15,16] and the Elfes Sensing Model (ESM) [17,18,19,20,21,22]. Studies applying the BSM assume that each sensor can detect the object falling in its sensing range. The probability of sensing can be expressed as a Boolean variable with a value of either true (one) or false (zero), depending on whether or not the event location is in the sensor’s sensing range. The BSM model is simple, but it is not practical as it does not consider interference and other environmental conditions. However, the sensing range in the real application is not a perfect disc due to the physical characteristic of sensing hardware and the interferences. In general, the actual sensing range of a sensor is smaller than the considered perfect disc and hence introduces a coverage hole when a coverage algorithm applies the BSM as its sensing model. On the contrary, the ESM is different from the BSM. It assumes that the detection of a target within the sensing range is a probabilistic value, which depends on the distance between the sensor and the target. The ESM can estimate the sensing capability of each sensor with a higher accuracy. Hence, it can obtain higher surveillance quality. This paper applies the ESM to construct a defense barrier.

Some other studies have applied the ESM to cope with the coverage problem. Dong et al. [17] proposed a centralized mechanism, which applied solar-powered sensors with adjustable sensing radii to construct the defense barrier. Compared with the mechanism proposed in [17], this paper proposed a distributed mechanism JSQE, which has better scalability and flexibility. Xu. et al. [21] applied the ESM to cope with the target coverage problem, aiming to minimize the total energy cost while satisfying the requirements of both coverage and connectivity. However, the mechanism proposed in [21] is not suitable for barrier coverage, which is the investigated issue of this paper. Fan et al. [22] focused on providing a cost-efficient directional barrier construction method, which aimed to form barriers and prolong network service lifetime. However, the surveillance quality was not considered [22]. In contrast to [22], this paper aims at constructing a barrier to guarantee the user-required surveillance quality.

Regarding the algorithm, plenty of existing studies of barrier coverage can be further divided into centralized [13,14,17] and distributed [15,16] approaches. The centralized mechanisms assume that the sink node is aware of the location of all sensors and executes an algorithm to determine the set of working sensors. To achieve this, the sink node needs to collect the reports from sensor nodes and makes a decision according to these reports. The big challenge of centralized approaches is that they increase communication overheads for obtaining the locations of all sensor nodes and then reporting the decision results to all sensors. These overheads consume more energy from sensors in advance. Another common challenge of centralized mechanisms is that they lack scalability, which is a key factor that should be considered in WSNs. To reduce the traffic overheads and energy consumption and improve the scalability of WSNs, some other studies have proposed distributed approaches, which allow each sensor to execute the monitoring work based on its local calculation. One big challenge of these algorithms is to make a decision without global information. In general, the surveillance quality increases with the number of working sensors. Therefore, another big challenge is to guarantee monitoring quality while reducing the number of working sensors to save energy.

This paper proposes a distributed Joint Surveillance Quality and Energy Conservation mechanism (JSQE), aiming to dynamically wake up the minimal number of working sensors to guarantee the required monitoring quality of the barrier boundary. In the proposed mechanism, the boundary curve is initially partitioned into several line segments to simplify the complexity of the investigated problem. Then, each sensor locally calculates its contribution to the monitoring quality. The sensor that has the largest contribution wakes up to join the monitoring work. The following presents the main contributions of this paper.

(1) Guaranteeing the predefined surveillance quality of the boundary barrier

The proposed JSQE mechanism guarantees the surveillance quality of the boundary barrier. This is achieved by identifying and improving the weakest monitoring quality round by round.

(2) Lower number of working sensors

In the proposed mechanism, the sensor that has the largest contribution to monitoring quality wakes up. This also implies that the number of working sensors can be reduced. Compared with existing works [15,17], the proposed mechanism wakes up lower numbers of working sensors.

(3) Scalability due to adopting the distributed approaches

The operations designed in the proposed JSQE are totally distributed. Each sensor locally calculates the contribution of monitoring quality and makes a decision. Adopting the distributed approaches can reduce the overheads of maintaining networks, such as network flooding and centralized operations. As a result, the efficiency of the proposed algorithm can be guaranteed even as the network grows, improving the scalability of the WSNs.

(4) Realistic

Compared with the existing studies [14,15,16] which apply BSM as the sensing model, this paper applies the ESM, which considers the physical characteristics of sensors and the interference of the external environment. Hence, the proposed JSQE mechanism is more realistic in the calculation of surveillance quality.

The remainder of this paper is organized as follows: Section 2 introduces the related work. Section 3 describes the network environment and problem formation. Section 4 details the proposed JSQE algorithm. Section 5 presents the simulation results, while Section 6 reviews the conclusions of this paper.

## 2. Related Work

This section presents the existing works related to the barrier coverage problem in WSNs. In the literature, plenty of studies have been developed for the construction of a defense barrier. These studies can be mainly classified into two categories: centralized and distributed approaches.

### 2.1. Centralized Approaches for Barrier Coverage

Chen. et al. [13] presented the concept of local barrier coverage, which can provide global barrier coverage in thin belt regions. They proposed a localized sleep–wake algorithm, aiming to prolong the network lifetime. Saipulla et al. [14] proposed a line-based sensor deployment strategy, which outperformed that of the Poisson point process in the monitoring region. Then, they further presented an efficient algorithm that adopts mobile sensors to fill gaps along the deployed line, aiming to improve barrier coverage. However, the long distance of movement leads to the energy consumption of the sensors. Dong et al. [17] introduced a centralized mechanism, aiming to improve the surveillance quality of a given boundary curve and to maintain the perpetual network lifetime. However, the centralized approaches [13,14,17] require the collection of messages from all sensors to the sink node, leading to low scalability and significant energy consumption. In addition, previous works [13,14] have designed centralized algorithms by applying the BSM model. However, the BSM model cannot accurately present physical characteristics, such as interference and other environmental conditions.

### 2.2. Distributed Approaches for Barrier Coverage

Weng et al. [15] first presented the Cover Adjacent Net (CA-Net) to simplify the problem of k-barrier coverage, aiming to reduce computational complexity. Based on the CA-Net, the distributed mechanism was presented to construct the maximum number of distinct k-barriers by using the minimum number of sensors. Xu et al. [16] proposed an efficient distributed algorithm that wakes up the minimal number of visual sensors to form a disjoint full-view barrier. The given region was first partitioned into a set of grids. Then, each visual sensor checked if its neighboring grids satisfied the full-view coverage. Finally, a locally weighted graph was formed by using the relationships among the girds meeting the full-view coverage. Based on the weighted graph, full-view barriers can be constructed. However, the above-mentioned existing works applied the BSM model, which cannot reflect the practical environment. This paper proposes a distributed mechanism, JSQE, by adopting the ESM model, which aims to maximize the weakest sensing quality of the barrier boundary while satisfying the user-defined monitoring quality with the minimum number of awake sensors. Table 1 simply compares these works with the proposed algorithm.

## 3. Network Environment and Problem

This section first introduces the network environment and assumptions in wireless sensor networks. Then, the sensor sensing model and the problem formulations are presented.

### 3.1. Network Environment

Assume that there is a barrier boundary *L* existing between the two countries. This boundary can also be the dividing line between dangerous and safe regions or between prohibited or open areas. Let *L* be a curve that can be modeled by function f(x) and can be contained in a minimal rectangle *R.* The left and right boundaries of *R* are denoted by $W_{l}$ and $W_{r}$, respectively. A set of sensors, $S=\left\{s_{1},s_{2},s_{3},\ldots \ldots ,s_{n}\right\}$, is randomly deployed in *R*. Let $\left(x_{i},y_{i}\right)$ denote the location of sensor $s_{i}$. Each sensor $s_{i}$ is assumed to have a unique ID and to be aware of its own location. All sensors are assumed to be clock synchronized. The sensing and communication radiuses are denoted by $r_{s}$ and $r_{c}$, respectively, where $r_{c}=2r_{s}$. Through the exchanges of beacons with one-hop neighbors, each sensor can collect the messages, including the IDs and locations of its neighbors.

### 3.2. Sensing Model

**Figure 1:**
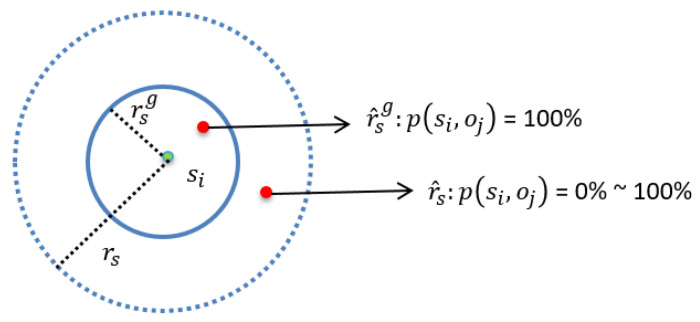
The applied Elfes Sensing Model.

This section introduces the ESM (Elfes Sensing Model) of the given wireless sensor networks. The sensing range of each sensor can be divided into two regions, denoted by ${\hat{r}}_{s}$ and ${\hat{r}}_{s}^{g}$, respectively. As shown in Figure 1, the probability of sensing is 100% if the object falls in the region ${\hat{r}}_{s}^{g}$. When the object falls in the region ${\hat{r}}_{s}$, the sensing probability decreases along with the increase in the distance between the sensor and the object.

Consider a point, v, in the region ${\hat{r}}_{s}$, and the coordinates of point v are $\left(x_{v},y_{v}\right)$. Let $p(s_{i},v)$ denote the sensing probability of point v detected by $s_{i}$, and $d(s_{i},v)$ denote the distance between sensor $s_{i}$ and point v. The following Equation (1) represents the relations between sensing probability p and distance d.

(1) $$p(s_{i},v)=\{\begin{array}{ll} 1 & d(s_{i},v)\leq r_{s}^{g}\\ e^{-\lambda (d(s_{i},v)-r_{s}^{g})}{}^{{}^{\gamma }} & r_{s}^{g}<d(s_{i},v)\\ 0 & d(s_{i},v)\geq r_{s} \end{array}<r_{s}$$

In Equation (1), the distance $d(s_{i},v)$ can be obtained through the following computation.

(2) $$d(s_{i},v)=\sqrt{{\left(x_{i}-x_{v}\right)}^{2}+{\left(y_{i}-y_{v}\right)}^{2}}$$

The λ and γ are the path-loss exponents of the sensing signal strength, which are adjusted by the different physical properties of sensors. In the case that point v satisfies the condition $r_{s}^{g}<d(s_{i},v)<r_{s}$, the detection probability $p(s_{i},v)$ of point v decreases with the reduction in the value of $d(s_{i},v)$.

### 3.3. Problem Formulation

Given a predefined monitoring quality ρ, the ρ-guaranteed barrier coverage problem aims to construct a defense barrier by scheduling a minimal set of working sensors, which are denoted by $\hat{S}$, such that any path crossing the boundary can be detected by the working sensors with a probability of at least ρ. In case the number of deployed sensors is not enough to support the ρ-guaranteed barrier coverage, the set of working sensors should guarantee that the monitoring quality of the barrier boundary can be maximized, while the number of sensors can be minimized. Let h represent the number of sensors in $\hat{S}$. The activation sensors $\hat{S}$ can be represented by Equation (3).

(3) $$\hat{S}=\left\{{\hat{s}}_{1},{\hat{s}}_{2},\ldots \ldots {\hat{s}}_{h}\right\},\hat{S}\subset S$$

**Figure 2:**
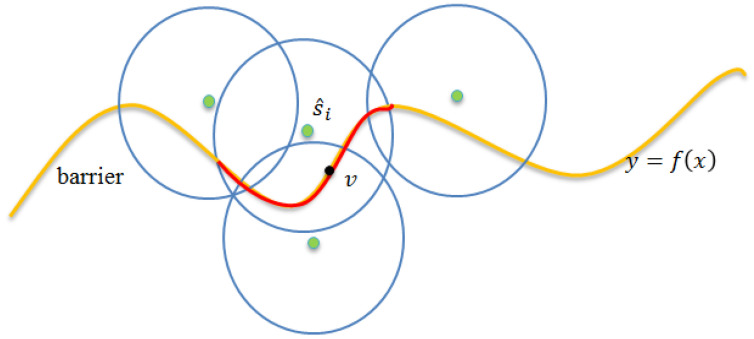
A scenario of the sensor set $\lambda_{v}$.

Figure 2 depicts the considered scenario. As shown in Figure 2, the sensing range of sensor ${\hat{s}}_{i}$ covers the segment marked with red ink of the boundary curve. For any point, say v, it can be monitored by sensor ${\hat{s}}_{i}$: that is, any intruder that crosses point v from south to north can be detected by sensor ${\hat{s}}_{i}$ using a probability. Let $\lambda_{i,v}$ denote the monitoring quality of point v contributed by sensor ${\hat{s}}_{i}$. Herein, we notice that the monitoring quality is a probability value as the probability sensing model is applied. Let the distance between sensor ${\hat{s}}_{i}$ and point v be represented by $d({\hat{s}}_{i},v)$. The following Equation (4) evaluates the value of $\lambda_{i,v}$, by applying the sensing model proposed in Equation (1).

(4) $$\lambda_{i,v}=p({\hat{s}}_{i},v)$$

Let $\lambda_{v}$ denote the probability of point v monitored by sensors in $S_{v}$. Let $S_{v}\subset S$ denote the set of sensors that can cover point v, and $\lambda_{v}$ denote the detection probabilities that the event of point v can be monitored by sensors in $S_{v}$. The weakest monitoring quality of any point in L, denoted by $\hat{\lambda }$, can be presented as

(5) $$\hat{\lambda }=\underset{v\in L}{\min}\lambda_{v}$$

This paper aims to maximize the weakest sensing quality of the barrier boundary while satisfying the minimum number of sensors. Equation (6) formulates the major goal of this paper.

(6) $$Objective\;Min(\rho ,\max imize(\hat{\lambda }))$$

When the major goal is achieved, the monitoring quality of the weakest segment has been maximized. This means that the quality of barrier coverage cannot be further improved even when a sensor wakes up. Therefore, the second goal is energy conservation, which aims to minimize the number of sensors that wake up. Equation (7) reflects this sub-goal:

(7) $$Minimize(\hat{n})$$

The goal is given in Equations (6) and (7), which should satisfy some constraints. The first is *the working state constraint*, which requires each sensor to remain in either a sensing or a sleeping state.

(1) *Working State constraint:*

(8) $$r_{i}{}^{sen}+r_{i}{}^{slp}=1,r_{i}{}^{sen},r_{i}{{}^{sl}}^{p}\in \left\{0,1\right\},\forall i$$

Let Boolean variables $r_{i}^{sen}$ and $r_{i}^{slp}$ denote sensing and sleeping states, respectively. Equation (8) reflects this constraint.

(2) *Sensor energy constraint:*

(9) $$e_{i}{}^{rem}\geq \frac{T}{t}*e_{t}{}^{\sec},\forall i$$

Let T denote the period of time required to monitor the barrier boundary. Recall that the time can be partitioned into several equal-length time units, denoted by t. Let $e_{t}^{\sec}$ denote the energy consumptions of any sensor staying in a sensing state for a time unit, t. Let $e_{i}^{rem}$ denote the remaining energy of sensor $s_{i}$. To ensure that the monitoring task can be executed over T, Equation (9) proposes that the battery energy of each sensor $s_{i}$ should be large enough to support the energy consumption.

(3) *Continuous constraint:*

For any sensor,$s_{i}\in \hat{S}$, there exists at least one sensor, $s_{j}\in \hat{S}$, such that the following condition is satisfied.

(10) $$s_{i}{}^{sen}\cap s_{j}{}^{sen}\neq \emptyset$$

Let $s_{i}^{sen}$ denote the sensing range of the *i*th sensor in $\hat{S}$. Equation (10) guarantees that there is no coverage hole existing in the constructed barrier.

(4) *Boundary constraint:*

(11) $$s_{leftmost}^{sen}\cap W_{l}\neq \emptyset \wedge s_{rightmost}^{sen}\cap W_{r}\neq \emptyset$$

Equation (11) shows that the sensing range of the leftmost sensor, which is denoted by $s_{leftmost}^{sen}$, should be overlapped with the left boundary of rectangle *R* (i.e.,$W_{l}$), and the sensing range of the rightmost sensor, denoted by $s_{rightmost}^{sen}$, should be overlapped with $W_{r}$.

The next section presents the proposed JSQE algorithm, which aims to achieve the goal given in Equations (6) and (7) while satisfying constraints Equations (8)–(11).

## 4. Joint Surveillance Quality and Energy Conservation (JSQE) Algorithm

The proposed JSQE is a distributed algorithm, which mainly consists of four phases: Boundary Curve Partitioning Phase, Basic Contribution Evaluation Phase, Collaborative Contribution Evaluation Phase, and Terminating Phase. In the Boundary Curve Partitioning Phase, a given barrier boundary,L, is partitioned into a set of equal-sized segments to simplify the barrier coverage problem. Each sensor in the Basic Contribution Evaluation Phase aims to independently calculate the monitoring quality of line segments it contributes. Based on the calculated contribution, the sensor determines its sleeping time. This can wake up the sensor with the largest contribution. Any sensor that stays in sleeping mode but learns that some neighbors have woken up should switch to the next phase, the Collaborative Contribution Evaluation Phase. The sensor staying in the Collaborative Contribution Evaluation Phase aims to calculate its contribution to the weakest segment. The calculation of the contribution should take into consideration the collaborative sensing of some common segments. The sensor that has the largest contribution to the weakest segment will be awake.

If any sensor that stays in sleeping mode determines that its contribution to the weakest segment is small enough, it should switch to the Terminating Phase. The sensor in the Terminating Phase does not participate in the monitoring task and stays in sleeping mode to save electricity.

The following presents the details of each phase of the proposed JSQE.

### 4.1. Boundary Curve Partitioning Phase

**Figure 3:**
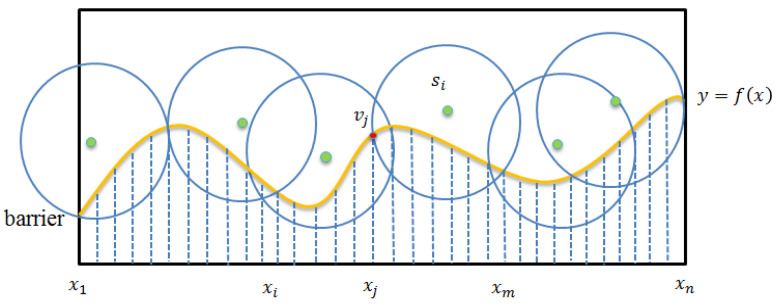
Segment-based partition.

This section presents the details of the Boundary Curve Partitioning Phase. The main purpose of this phase is to partition the boundary curve into several equal-length line segments. To better present the details, the following provides some formal definitions. Assume that the boundary curve can be mapped to a geographic two-dimensional plan, and it can be modeled by function $y=f(x),x_{1}\leq x\leq x_{n}$, where $x_{1}$ and $x_{n}$ denote the x coordinates of the leftmost and rightmost points of the boundary curve, respectively. In general, the direction for partitioning the boundary is taken as the direction that is perpendicular to the boundary. Without the loss of generality, this paper simply assumes that the boundary is horizontal. To facilitate the evaluation of the monitoring contribution of each sensor, as shown in Figure 3, we partition the boundary curve into a set of equal-sized *n*-line segments. Let $L[x_{a}:x_{b}]$ denote a sub-curve of the boundary curve, starting from *x*-coordinate $x_{a}$ and ending at *x*-coordinate $x_{b}$. The boundary curve can be presented by set $L=\left\{l_{k}|l_{k}=\left[x_{k}:x_{k+1}\right],1\leq k\leq n-1\right\}$ or simplicity $L\left[x_{1}:x_{n}\right]$.

**Figure 4:**
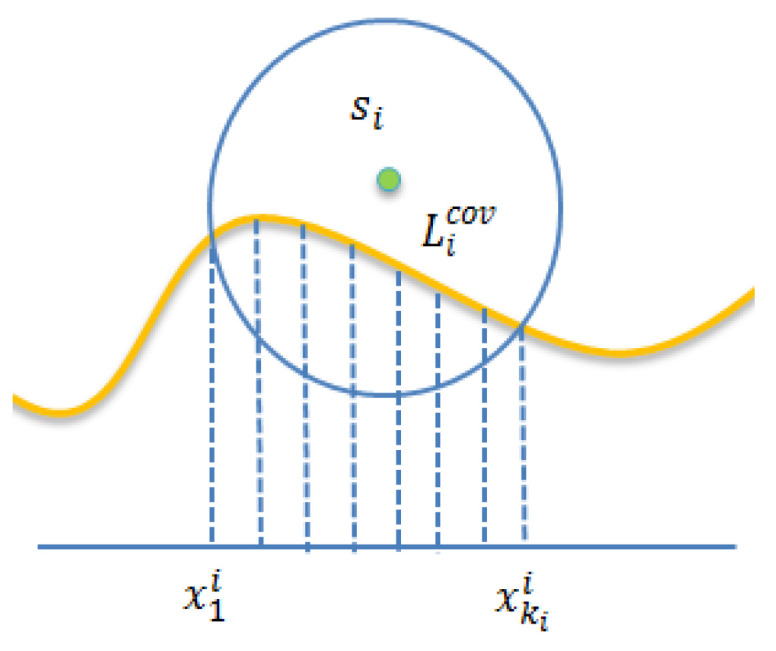
The covered segment of.

For simplicity, we assume that each sensor can cover several complete line segments. Thus, the covered segment of sensor $s_{i}$ is denoted by $L_{i}^{\mathrm{cov}}$, as shown in Figure 4. Assume that the length of $L_{i}^{\mathrm{cov}}$ is $k_{i}$. The covered segment of sensor $s_{i}$ can be represented as shown in Equation (12).

(12) $$L_{i}^{\mathrm{cov}}=L[x_{start}^{i}:x_{end}^{i}]=\left\{\left[x_{1}^{i}:x_{2}^{i}\right],\left[x_{2}^{i}:x_{3}^{i}\right],\ldots \ldots \left[x_{k_{i-1}}^{i}:x_{k_{i}}^{i}\right]\right\}$$

### 4.2. Basic Contribution Evaluation Phase

In this phase, each sensor independently calculates its contribution to monitoring quality. According to its own contribution, each sensor determines its waiting time. In order to wake up a minimal number of sensors to monitor the barrier boundary, the sensor with the largest contribution should wake up the earliest and then broadcast the join-monitoring message, which includes the ID and location of that sensor in a distributed manner. The following presents the detailed calculation of the contribution of each sensor.

Initially, all sensors stay in the sleeping state. Herein, we notice that each sensor $s_{i}\subset S$ is aware of its coordinates $\left(x_{i},f(x_{i})\right)$. Consider any point v that lies in the segment $L_{i}^{\mathrm{cov}}$. Assume that the coordinates of point v are $\left(x_{v},f(x_{v})\right)$. The following introduces how to calculate the monitoring quality of point *v* contributed by $s_{i}$. Let $p(s_{i},v)$ denote the monitoring quality of point v contributed by sensor $s_{i}$. Let notation d(u,v) denote the distance of two points, u and v. The distance between sensor $s_{i}$ and v can be denoted by d(u,v), whose value can be obtained by Equation (13).

(13) $$d(s_{i},v)=\sqrt{{\left(x_{i}-x_{v}\right)}^{2}+{\left(f(x_{i})-f(x_{v})\right)}^{2}}$$

By applying the probabilistic model, as shown in Equation (1), the value of $p(s_{i},v)$ can be calculated through Equation (14).

(14) $$p(s_{i},v)=\{\begin{array}{cc} 1 & d(s_{i},v)\leq r_{s}^{g}\\ e^{-\lambda {\left(d(s_{i},v)-r_{s}^{g}\right)}^{\gamma }} & r_{s}^{g}<d(s_{i},v)<r_{s} \end{array}$$

The total contribution of sensor $s_{i}$, which is denoted by $c_{i}$, for its covered segment, $L_{i}^{\mathrm{cov}}$, can be evaluated by applying Equation (15).

(15) $$c_{i}=\int_{x_{1}^{i}}^{x_{k_{i}}^{i}}p(s_{i},v)dx,v\in L_{i}^{\mathrm{cov}},s_{i}\in S$$

As we aim to wake up as few sensors as possible, the policy design for waiting time is that the sensor with the largest contribution should wake up the earliest. Equation (16) depicts the calculation of the waiting time.

(16) $$t_{i}=\frac{1}{c_{i}},\forall i$$

**Figure 5:**
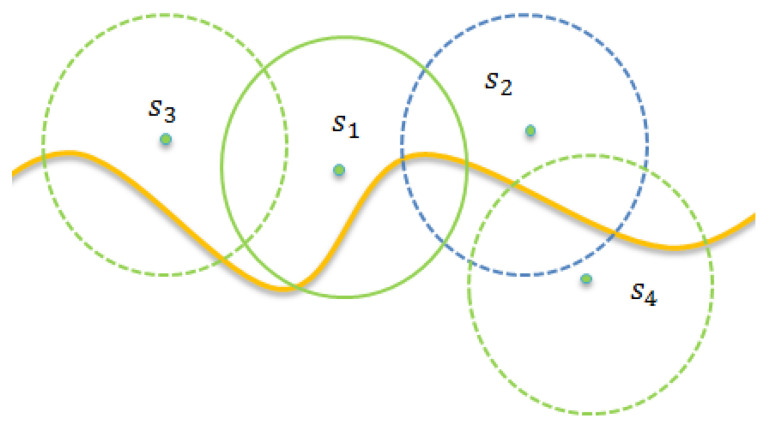
An example to show that sensor $s_{1}$ wakes up earlier than sensor $s_{2}$.

Figure 5 provides an example to show that sensor $s_{1}$ wakes up earlier than sensor $s_{2}$. As shown in Figure 5, assume that the contribution of any sensor $s_{1}$ is $c_{1}$. As the value of $c_{1}$ is larger than that of $c_{2}$, the waiting time of sensor $s_{1}$ is shorter than that of sensor $s_{2}$, according to Equation (16). When sensor $s_{1}$ finishes its waiting time, it wakes up and broadcasts the join-monitor message, which contains its ID = $s_{1}$ and physical location $\left(x_{1},f(x_{1})\right)$ in a distributed manner.

Upon receiving the join-monitor message from a neighbor, each sensor learns the fact that its neighbor has been awake. The neighboring sensors should check whether or not they should switch to the next phase.

The following presents the criteria for the switching phase. A sensor, say $s_{j}$, is said to be a loser of sensor $s_{i}$ if it satisfies the following two conditions. Similarly, sensor $s_{i}$ is said to be a winner if it joins the monitoring task.

Phase switching criteria:

(1) Sensor $s_{j}$ neighbors $s_{i}$.
(2) The covered segments of $s_{i}$ and $s_{j}$ are overlapped, that is, the following condition holds.

(17) $$L_{i}^{\mathrm{cov}}\cap L_{j}^{\mathrm{cov}}\neq \emptyset$$

Each sensor that stays in sleeping state but hears the join-monitor message should further check if it satisfies the phase switching criteria. If this is the case, the sensor switches to the Collaborative Contribution Evaluation Phase.

### 4.3. Collaborative Contribution Evaluation Phase

The collaborative contribution evaluation phase will be initiated if the sensor receives the join-monitor message and satisfies the phase switching criteria. In this phase, each sensor should recalculate its contribution to its covered segment if it has an overlapped segment with some awake sensors. Recall the major goal of the considered barrier problem is to maximize the monitoring quality of the weakest point. Therefore, the contribution calculation only cares about monitoring contribution to the weakest point, rather than the monitoring contribution to the whole covered segment. The sensor that has the largest contribution to the weakest line segment should be awake and join the monitoring task.

**Figure 6:**
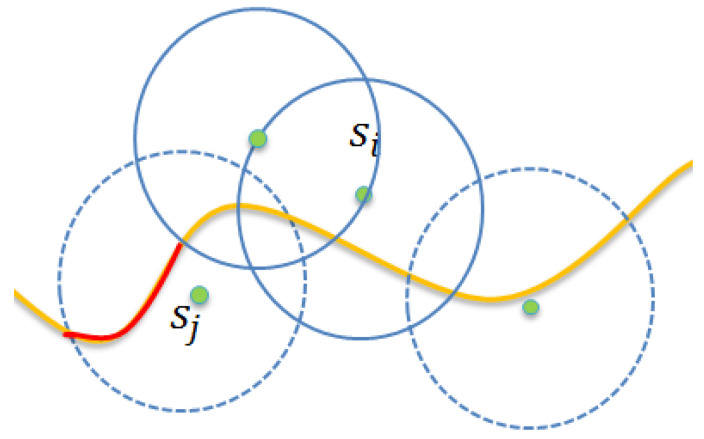
Classified into two types, denoted by $L_{j}^{no\mathrm{cov}ered}$ marked with red ink and $L_{j}^{overlapped}$, respectively.

Assume that sensor $s_{i}$ is awake and has joined the monitoring tasking. Assume that sensor $s_{j}$ is the loser due to receiving the join-monitoring message from $s_{i}$. When sensor $s_{j}$ switches to the collaborative contribution evaluation phase, it should recalculate the contribution of the overlapped segment. Let the covered segment $L_{j}^{\mathrm{cov}}$ be classified into two types according to whether or not the segment is overlapped segment. As shown in Figure 6, let $L_{j}^{no\mathrm{cov}ered}$ denote the no-covered segment marked with red ink by the sensor $s_{j}$. Similarly, let $L_{j}^{overlapped}$ denote the overlapped segment that is commonly covered by the sensor $s_{j}$ and awake sensors. That is, we have

(18) $$L_{j}^{\mathrm{cov}}=L_{j}^{no\mathrm{cov}ered}\cup L_{j}^{overlapped}\;$$

Let $p_{m}^{no\mathrm{cov}ered}$ denote the monitoring probability of each line segment $l_{m}\in L_{j}^{no\mathrm{cov}ered}$. According to the ESM, the value of $p_{m}^{no\mathrm{cov}ered}$ can be obtained by Equation (19).

(19) $$p_{m}^{no\mathrm{cov}ered}=0$$

Assume that $S^{l_{q}}\subset S$ denotes the set of awake sensors that can cover line segment $l_{q}\in L_{j}^{overlapped}$. It is obvious that $s_{j}\notin S^{l_{q}}$. The calculation of the contribution of sensor $s_{j}$ to line segment $l_{q}$ should take into consideration collaborative monitoring. Let ${\tilde{p}}_{q}^{overlapped}$ denote the probability that none of the awake sensors in $S^{l_{q}}$ can monitor line segment $l_{q}$. For simplicity, the leftmost point, say $v_{q}$, of line segment $l_{q}$, represents line segment $l_{q}$, that is, the monitoring probability of $v_{q}$ presents the monitoring probability of segment $l_{q}$. We have

(20) $${\tilde{p}}_{q}^{overlapped}=\prod_{s_{k}\in S^{l_{q}}}\left(1-p(s_{k},v_{q})\right)$$

Let $p_{q}^{overlapped}$ denote the probability that any awake sensor in $S^{l_{q}}$ can monitor line segment $l_{q}$. According to Equation (20), the value of $p_{q}^{overlapped}$ can be calculated as shown in Equation (21).

(21) $$p_{q}^{overlapped}=1-{\tilde{p}}_{q}^{overlapped}=1-\prod_{s_{k}\in S^{l_{q}}}\left(1-p(s_{k},v_{q})\right)$$

Equation (21) depicts the probability that any sensor in $S^{l_{q}}$ can monitor line segment $l_{q}$. However, we need to evaluate the monitoring probability contributed by sensor $s_{j}$. Let Φ(I) denote the probability that any sensor in set I can monitor line segment $l_{q}$. It is obvious that we have

(22) $$\Phi (S^{l_{q}})=p_{q}^{overlapped}$$

Equation (23) represents the increased probability contributed by the single sensor, $s_{j}$, if sensor $s_{j}$ wakes up and cooperatively monitors line segment $l_{q}$.

(23) $$\Phi (S^{l_{q}}\cup s_{j})-\Phi (S^{l_{q}})=\left[1-\prod_{s_{k}\in S^{l_{q}}\cup s_{j}}\left(1-p(s_{k},v_{q})\right)\right]-\left[1-\prod_{s_{k}\in S^{l_{q}}}\left(1-p(s_{k},v_{q})\right)\right]$$

Given that the set of awake sensors is denoted by $S^{l_{q}}$, let $l_{j}^{weakest}$ denote the line segment with the weakest monitoring probability and $p_{j}^{weakest}$ denote its monitoring probability. We have

(24) $$l_{j}^{weakest}=\left\{ \begin{array}{l} l_{h},l_{h}\in L_{j}^{no\mathrm{cov}ered},1\leq h\leq k_{i}\\ \underset{l_{h}\in L_{j}^{\mathrm{cov}}}{\arg}\min\Phi (S^{l_{h}}),S^{l_{h}}\neq \emptyset \end{array}\right.\;p_{j}^{weakest}=\Phi (S^{l^{weakest}})$$

As we aim to maximize the monitoring quality of the weakest line segment while reducing the number of awake sensors, the waiting times of sensors should be decreased alongside their contributions for the weakest line segment covered by $s_{j}$. Let $c_{j}$ denote the contribution of the single sensor, $s_{j}$, for the weakest line segment. The value of $c_{j}$ can be calculated as shown in Equation (25).

(25) $$c_{j}=\Phi (S^{l^{weakest}}\cup s_{j})-\Phi (S^{l^{weakest}})$$

Thus, the waiting time of each sensor $s_{j}$ can be presented as

(26) $$t_{j}=\frac{1}{c_{j}\times \frac{1}{p_{j}^{weakest}+1}}$$

Upon completing the waiting time, sensor $s_{j}$ takes part in the monitoring work and, therefore, plays the role of the *winner*. All the other sensors neighboring the *winner*, which still stay in a sleeping state, are *losers* of the $s_{j}$. Each *loser*, say, $s_{w}$, should recalculate its own contribution to the overlapped segment and then reset its waiting time accordingly. Each *loser* should repeatedly execute the Collaborative Contribution Evaluation Phase to maximize the monitoring quality. The *loser* will switch to the Terminating Phase until the following condition is satisfied.

(27) $$c_{w}\leq \sigma$$

where σ is the predefined contribution threshold, that is, the monitoring quality of the weakest line segment cannot be significantly improved, even though the *loser* wakes up to participate in the monitoring task. 

### 4.4. Terminating Phase

Each sensor switches to the Terminating Phase and should stay in the sleeping state until some neighboring working sensors run out of energy. The sleeping sensor should switch to the Collaborative Contribution Evaluation Phase to check if it should participate in the monitoring work.

In this section, the JSQE algorithm, which consists of four phases, is proposed for maximizing the weakest sensing quality of the given barrier boundary while reducing the number of awake sensors. Each sensor performs the proposed JSQE and locally determines whether it should participate in the monitoring task, and plays the role of the working sensor. Finally, the set of working sensors is the output of the proposed JSQE algorithm. In the next subsection, the formal algorithm is presented.

### 4.5. The Proposed JSQE Algorithm

This subsection presents the JSQE algorithm to summarize the operations presented in the previous subsection.

The overall distributed JSQE algorithm is shown in Algorithm 1. Each step will be performed locally by sensor $s_{i}$. In phase I, steps 1 and 2 evaluate the covered segment of sensor $s_{i}$ and the number of line segments covered by sensor $s_{i}$. In phase II, steps 3 to 5 calculate the contribution of each sensor $s_{i}$, then obtain the waiting time accordingly. Next, step 6 calls the *Wait*() procedure to reduce the waiting time in order to determine the role of sensor $s_{i}$. Steps 7 to 9 check whether or not the *loser* $s_{i}$ has an overlapped segment with its neighbor working sensor. The calculation of the contribution of the overlapped segment should take into consideration collaborative sensing. In phase III, steps 10 to 15 evaluate the line segment with the weakest monitoring probability covered by sensor $s_{i}$. Step 16 calculates its monitoring probability. Next, steps 17 and 19 calculate the contribution of the single sensor, $s_{i}$, and its waiting time. Step 20 calls the *Wait*() procedure to determine the role of sensor $s_{i}$. When sensor $s_{i}$ is a *loser*, steps 21 to 23 check if it satisfies the criteria to participate in the monitoring task. In phase IV, steps 24 to 27 express that the *loser* $s_{i}$ stays in a sleeping state until its neighbor working sensor runs out of energy. The *loser* $s_{i}$ then switches to phase III. Step 28 returns a set of all working sensors, ${\hat{S}}^{best}$, and finishes the JSQE executions.
**Algorithm 1**. Joint Surveillance Quality and Energy Conservation (JSQE)**Inputs:** A set of sensors, $S=\left\{s_{1},s_{2},\ldots \ldots ,s_{n}\right\}$. Notation (*x_i_*, *y_i_*) denotes the location of sensor $s_{i}$. The boundary curve can be modeled by function $y=f(x),\;x_{1}\leq x\leq x_{n}$, where $x_{1}$ and $x_{n}$ denote the *x* coordinates of the leftmost and rightmost points of the boundary curve, respectively. A partitioned boundary curve with *n* line segments.**Output:** The set of working sensors ${\hat{S}}^{best}$.**//Phase** I. **Boundary Curve Partitioning Phase//**
1. Sensor $s_{i}$ evaluates the covered line segments $L_{i}^{\mathrm{cov}}$ according to Equation (12);
2. Let $k_{i}$ denote the number of line segments covered by sensor $s_{i}$;
**//Phase** II. **Basic Contribution Evaluation Phase//**
3. Each sensor $s_{i}$ executes the following operations.
4. Evaluate its contribution $c_{i}$ according to Equation (15);
5. Set up its waiting time $t_{i}$ according to Equation (16);
6. Call $wait(t_{i})$;
7. **If** (The *loser*
$s_{i}$ has no overlapped segment with any neighboring working sensor)
8. Go to Step 6;
9. **End**
**If**
**//Phase** III. **Collaborative Contribution Evaluation Phase//**
10. **For each**
$l_{q}\in L_{i}^{no\mathrm{cov}ered}$
11. Evaluate $p_{m}^{no\mathrm{cov}ered}=0$;
12. $l_{i}^{weakest}=l_{q}$;
13. **End**
**for**
**14.** Evaluate $p_{q}^{overlapped}$ according to Equation (21);
15. $l_{i}^{weakest}=\underset{l_{q}\in L_{i}^{\mathrm{cov}}}{\arg}\min p_{q}^{overlapped}$;
16. Evaluate $p_{i}^{weakest}=\Phi (S^{l_{i}^{weakest}})$;
17. Let $\Phi (S^{l_{q}})=p_{q}^{overlapped}$;
18. Evaluate $c_{i}$ according to Equation (25);
19. Evaluate $t_{i}$ according to Equation (26); //set up waiting time
20. Call $wait(t_{i})$;
21. **If** ($c_{i}>\sigma$)//σ is the predefined contribution threshold
22. Goto 10;
**23. End**
**if**
**//Phase** IV. **Terminating Phase//**
24. Sensor $c_{i}$ stays in sleeping state;
25. **While** (listen()! = null)
26. Goto 10;//$s_{i}$ is a *loser* again
27. **EndWhile**
**28.** Return ${\hat{S}}^{best}$;//the set of working sensors
**//Procedure *Wait*()//**
Procedure *Wait*(Timer *t_i_*){
**While**(*listen*( )=**Null** or backoff time *t_i_* >0){
  Wait for one time slot;
  backoff time $t_{i}$--; }
 **EndWhile**
 **If** (backoff time $t_{i}$ = 0) {
  Wake up and set My_role = *winner*;
 End of Scheduling and switch to working state; }
**End**
**If**
 My_role = *loser*; }

The following describes the computing complexity of this algorithm. In phase I, the computing complexity of steps 1 and 2 is O(1). In phase II, each sensor evaluates its contribution and obtains the waiting time in steps 3 to 5. Thus, the computing complexity of steps 3 to 5 is O(1). In phase III, steps 10 to 13 evaluate the weakest line segment of the loser, $s_{i}$, which is not an overlapped segment. The computing complexity is O(*q*). Step 14 further evaluates the collaborative probability of the neighbors of any awake sensor. As the number of neighbors is *k*, the computing complexity of this step is O(*k*). Steps 15 and 16 evaluate the weakest line segment and its monitoring quality. The computing complexity of steps 15 and 16 are O(*q*) and O(*k*), respectively. Steps 17 to 19 calculate the contribution of the *loser*, $s_{i}$, for the weakest line segment and set up the waiting time. The computing complexity of steps 17 to 19 is O(1). In phase IV, the computing complexity of steps 24 to 27 is O(1). Therefore, the computing complexity of the JSQE algorithm is max(O(*k*), O(*q*)).

## 5. Simulation

This section presents the performance comparisons of the proposed JSQE against the existing top-down one-coverage barrier approach (TOBA) [15] and Boundary Surveillance mechanism with the adjustable sensing radius (BSAS) [17]. Based on the developed CA-Net (Cover Adjacent Net), the TOBA mechanism forms a k-barrier by combining multiple 1-barriers with the purpose of minimal energy consumption and maximal lifetime. The BSAS mechanism constructs the defense barriers with the sensor of the adjustable sensing radius, aiming to improve the surveillance quality of the barrier boundary and the utilization of sensors. The following first illustrates the simulation environment and then presents the simulation results.

### 5.1. Simulation Environment

**Figure 7:**
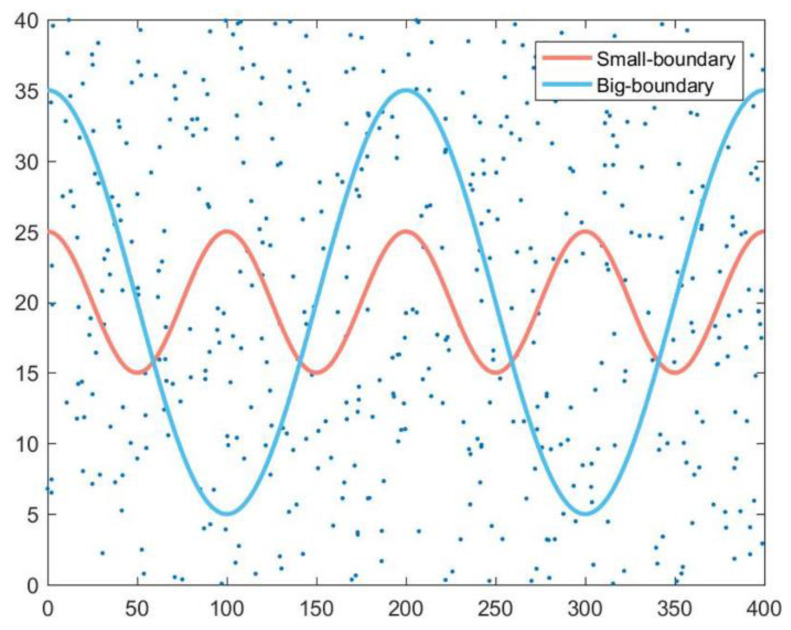
Small-boundary (S-b) and Big-boundary (B-b).

The simulation parameters are given in Table 2. MATLAB is used as the simulation tool in the experimental study. The sensor nodes are randomly deployed in the 400 m × 40 m monitoring area, as shown in Figure 7. The number of sensors is varied, ranging from 400 to 800. Each node is aware of its own location. The communication radius is twice the size of the sensing radius, which is set to 10m. The user-required monitoring quality, denoted by $Q_{req}$, is set to 0.3, 0.5, and 0.7. To further study the performance of the proposed JSQE mechanism, two different boundaries, namely, Small-boundary and Big-boundary, are considered, which are denoted by S-b and B-b, respectively.

### 5.2. Simulation Results

**Figure 8:**
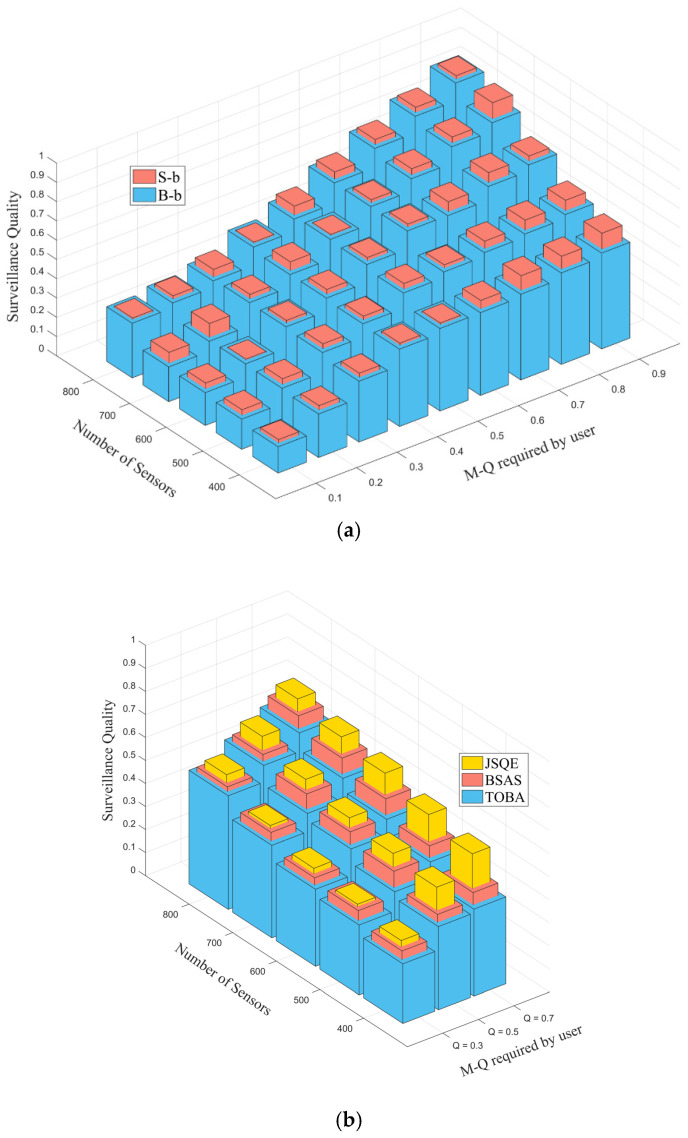
Performance comparison of boundary surveillance quality applying different $Q_{req}$ and varying the number of deployed sensors: (**a**) using JSQE to compare Small-boundary and Big-boundary; (**b**) using Small-boundary to compare JSQE, BSAS, and TOBA mechanisms.

Figure 8 investigates the boundary surveillance quality under different numbers of deployed sensors and different required quality, $Q_{req}$. As shown in Figure 8a, both the Small-boundary and Big-boundary are considered, and the surveillance quality of the proposed JSQE is measured. The performance of the Small-boundary is better than that of the Big-boundary in all cases. This occurs because the Small-boundary has a small amplitude, where the line segments are concentrated around the central barrier. As a result, the line segments can be easily covered by the deployed sensors, resulting in higher monitoring quality.

Figure 8b further compares the surveillance qualities of JSQE, BSAS, and TOBA mechanisms by varying the number of sensors and the quality requirement, $Q_{req}$. Three mechanisms have a similar trend in that the quality is increased with the increase in the number of deployed sensors and the required quality. This occurs because more sensors can participate in monitoring tasks when a large number of sensors are deployed. Furthermore, the proposed JSQE has a higher monitoring quality in the case of the low-density sensor deployment and high user-required quality $Q_{req}$, as compared with the other two mechanisms. The reason for this is that the proposed JSQE adopts the ESM model, which can reflect the physical parameters. It selects the sensor with the maximal contribution to improve the surveillance quality of the bottlenecked line segments.

**Figure 9:**
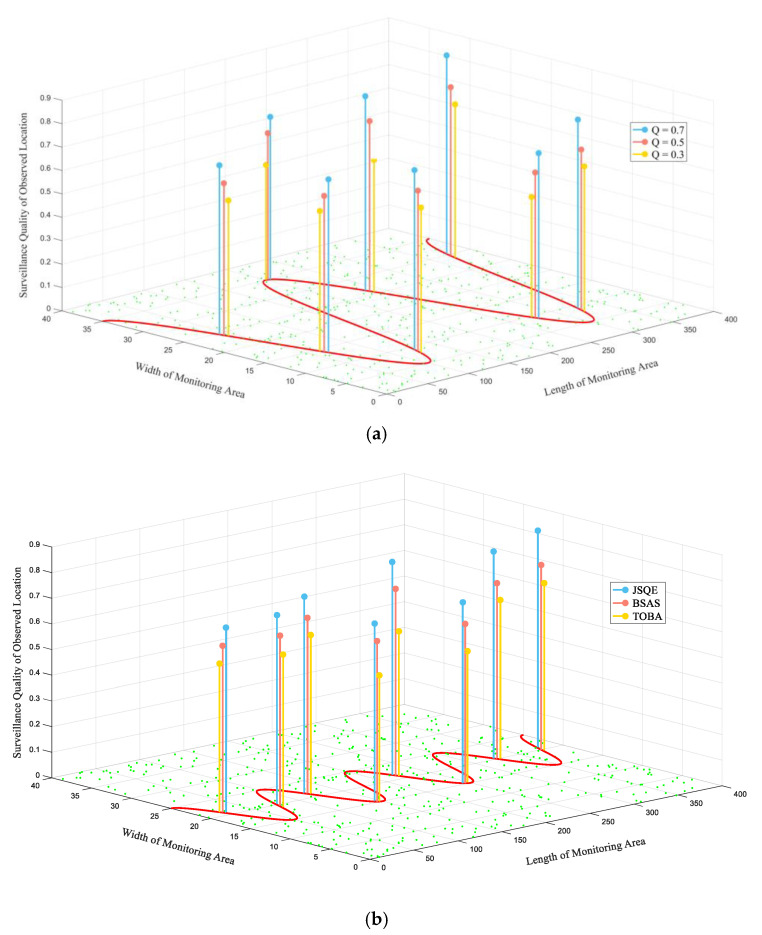
Performance comparison of surveillance quality by randomly selecting points: (**a**) different $Q_{req}$ of JSQE for Big-boundary; (**b**) different mechanisms for Small-boundary.

Figure 9 compares the surveillance qualities of the eight randomly selected points when 500 sensors are randomly deployed in a monitoring region. Figure 9a shows the surveillance quality of the eight selected points on the Big-boundary by applying the proposed JSQE. It is observed that the surveillance qualities of all selected points are higher than the user-required value $Q_{req}$. This implies that the proposed JSQE supports sufficient fairness of surveillance quality. This occurs because the selection policy of the JSQE mechanism selects the sensor with the largest contribution to cover the bottleneck segment, which can balance the surveillance quality of each line segment.

Figure 9b further compares the surveillance qualities of the proposed JSQE and existing BSAS and TOBA by using different boundaries. The required quality $Q_{req}$ is set to 0.7, while the settings of other parameters are identical to those of Figure 9a. In comparison, the proposed JSQE mechanism outperforms BSAS and TOBA in terms of the monitoring quality for those randomly selected points. The reason for this is that the existing TOBA mechanism aims to construct the k-barrier with a minimal number of working sensors. This policy might lead to the imbalanced monitoring quality of each line segment, as the line segment with the minimal quality might not be covered by the working sensors. In addition, the BSAS mechanism is a centralized approach, which consumes more energy to control overheads. Therefore, a lower amount of remaining energy results in lower surveillance quality, as compared with the proposed JSQE.

**Figure 10:**
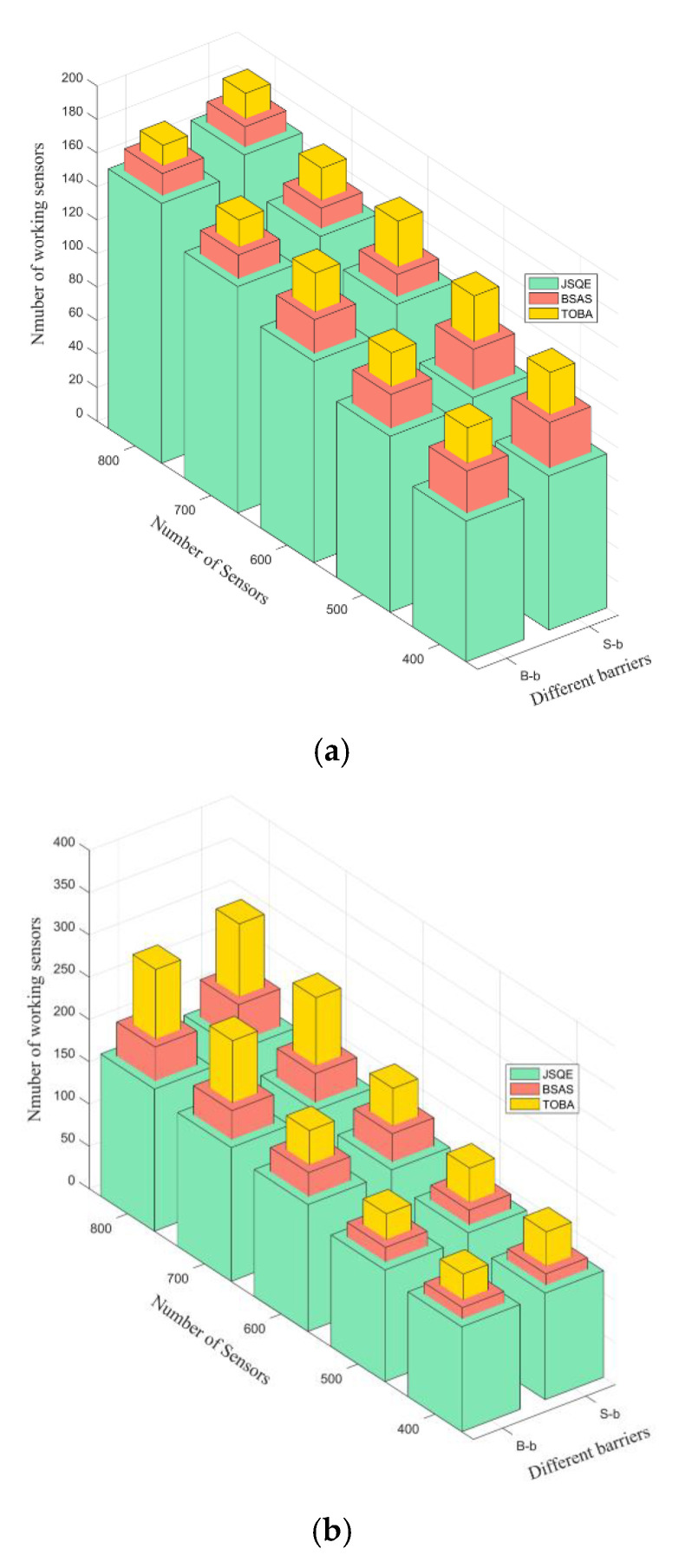
Comparison of the three mechanisms in terms of the number of working sensors in different values of $Q_{req}$: (**a**) $Q_{req}$ is set to 0.3; (**b**) $Q_{req}$ is set to 0.7.

Figure 10 compares the number of working sensors by applying the three mechanisms in the Small-boundary scenario. The number of deployed sensors ranges from 400 to 800. As shown in Figure 10, three mechanisms have a similar trend in that the number of working sensors is increased with the increase in the number of deployed sensors. This occurs because a large number of deployed sensors can provide more opportunities to find the appropriate sensors, which contribute better monitoring quality to satisfy the user-required quality $Q_{req}$. As shown in Figure 10a, in the case that the required quality $Q_{req}$ is equal to 0.3, three mechanisms have small differences in terms of the number of working sensors. This is because all three mechanisms have a similar trend in that fewer sensors can achieve the requirements of surveillance quality. Figure 10b shows that the proposed JSQE mechanism has better performance than BSAS and TOBA, especially in the scenario in which there is a high density of sensor nodes. This occurs because the proposed JSQE mechanism can exactly estimate the surveillance quality by applying the ESM. In addition, the JSQE selects more sensors with the largest contribution, such that the segment with the weakest surveillance quality can be significantly improved. For example, when the weakest surveillance quality is below quality $Q_{req}$ = 0.7, the JSQE mechanism only needs to wake up a few sensors to improve the weakest surveillance quality. On the contrary, the existing TOBA mechanism wakes up more sensors to construct the barrier, aiming to form the continuous cover set. The BSAS mechanism first schedules sensors with the farthest distance to the boundary barrier, aiming to improve the utilization of sensors. This results in a larger number of working sensors, as compared with the proposed JSQE.

**Figure 11:**
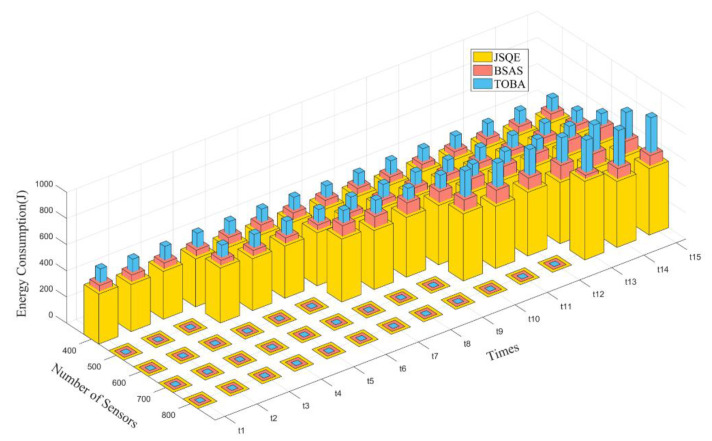
Comparison of three mechanisms in terms of energy consumption by increasing the number of sensors.

Assume that all sensors have the same total energy. As the working sensors run out of energy, we add 100 sensors over a fixed period. As shown in Figure 11, the three mechanisms have a similar trend in that the energy consumption increases is increased with the number of sensor nodes. The reason for this is that adding sensors allows for the energy consumption needed to construct the new barriers. The total energy consumption of the JSQE is lower than that of the other two mechanisms. This is because the JSQE schedules a smaller number of working sensors but satisfies the user-defined surveillance quality.

**Figure 12:**
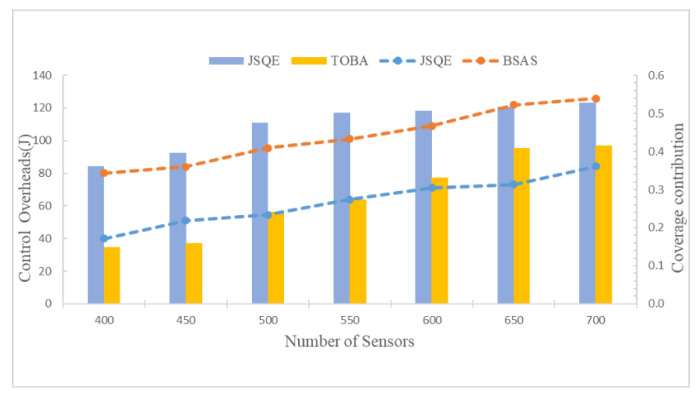
Comparison of JSQE, BSAS, and TOBA in terms of control overheads and coverage contribution by increasing the number of sensors.

Figure 12 compares the flexibilities of the JSQE, BSAS, and TOBA algorithms. The flexibility of an algorithm is observed by measuring the control overheads and the increment of coverage contribution when the number of deployed sensors is increased. In the experiment, fifty sensors are added every fixed period of time. The number of sensors is varied, ranging from 400 to 700. As shown in Figure 12, the proposed JSQE outperforms the BSAS in terms of control overheads. The reason for this is that the BSAS algorithm adopts a centralized approach, which increases significant control overheads for communication when 50 additional sensors are deployed. Meanwhile, the proposed JSQE has better performance than the TOBA in terms of the coverage contribution. By applying the JSQE, the newly scheduled sensors can accurately calculate their contributions and improve the weakest surveillance quality. However, the TOBA can increase its coverage only when the number of increased sensors is larger than a certain number. Thus, the JSQE is better than the BSAS and TOBA in terms of the scalability of the WSNs.

**Figure 13:**
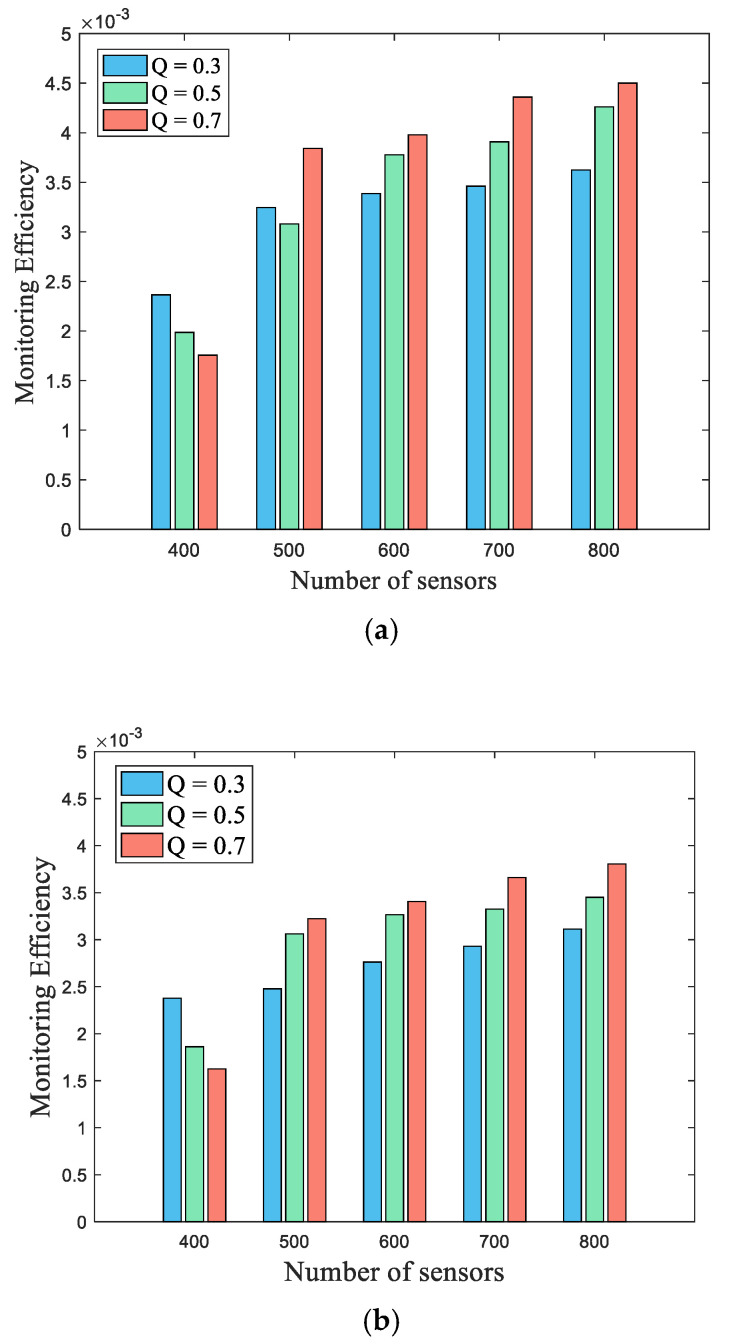
Comparison of the different user-required qualities $Q_{req}$ in terms of monitoring efficiency by varying the number of deployed sensors: (**a**) Small-boundary; (**b**) Big-boundary.

Figure 13 further examines the efficiency index, which considers two parameters, namely, the weakest surveillance quality and the number of working sensors. Let $N_{num}$ denote the total number of working sensors and $P_{weakest}$ denote the weakest monitoring quality of the barrier boundary. The monitoring efficiency, denoted by $M_{efficiency}$, is defined as shown in Equation (28).

(28) $$M_{efficiency}=\frac{p_{weakest}}{N_{num}}$$

A high-efficiency index indicates that the mechanism can obtain better monitoring quality by using fewer working sensors. In Figure 13, the monitoring efficiency is examined for the three required qualities $Q_{req}$ in two scenarios, where the number of deployed sensors is varied, ranging from 400 to 800. As shown in Figure 13, the monitoring efficiency at $Q_{req}$ = 0.7 is better than that of the other two required qualities, in the case that the number of sensors is larger than 500. This occurs because more appropriate sensors with better contributions can be selected in the case of a large number of sensors. Comparing Figure 13a,b, the performance in the Small-boundary scenario is better than that in the Big-boundary scenario in all cases. This occurs because the Big-boundary has a large amplitude, leading to the segments being widely distributed. Hence, the segments of the barrier cannot be easily covered by the sensors.

**Figure 14:**
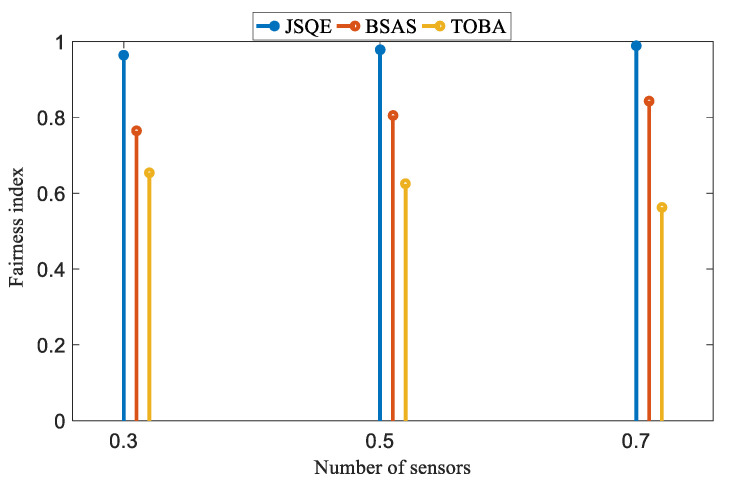
Performance comparison of JSQE, BSAS, and TOBA in terms of fairness index.

Figure 14 further investigates the fairness indices of the three compared mechanisms in the Small-boundary scenario. The user-required quality is set to 0.3, 0.5, and 0.7. The number of sensors is 500. The fairness index of monitoring quality, denoted by $T_{fairness}$, is defined as shown in Equation (29), where $p_{i}$ denotes the monitoring quality of each line segment and *n* denotes the number of line segments.

(29) $$T_{fairness}={\frac{\left(\sum_{i=1}^{n}p_{i}\right)}{n\sum_{i=1}^{n}p_{i}{}^{2}}}^{2}$$

In comparison, the proposed JSQE outperforms the other two mechanisms in terms of the fairness index. The key reason for this is that the TOBA does not consider the lowest monitoring quality, which impacts its fairness. The BSAS aims to maximize the utilization of sensor energy. This policy leads to low fairness when the deployed sensors are not balanced. The proposed JSQE finds the weakest line segment and selects the sensors with the largest contribution to improve the monitoring quality of the weakest line segment. Therefore, the fairness index of JSQE is close to 1.

**Figure 15:**
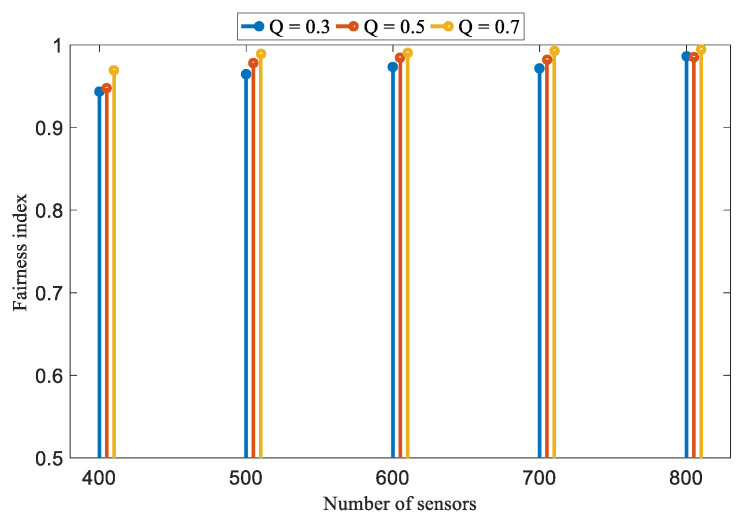
Performance comparison of different required qualities in terms of fairness index.

Figure 15 further examines the fairness index of the JSQE mechanism by varying the values of the required quality in the Small-boundary scenario. The number of sensors is varied, ranging from 400 to 800. As shown in Figure 15, the fairness index of monitoring quality increases with the increase in the number of deployed sensors. This occurs because more sensors can provide more opportunities for sensors to cooperatively monitor the bottleneck line segment, which has the lowest monitoring quality.

## 6. Conclusions

This paper proposes a distributed barrier coverage mechanism, JSQE, which aims to dynamically wake up the minimal number of working sensors to guarantee the required monitoring quality of the barrier boundary. The proposed JSQE initially partitions the boundary curve into several line segments. Then, it applies the ESM to calculate the sensing probability of each sensor, identify the weakest line segments, and schedule the sensor with the maximal contribution in advance to improve the weakest line segment. The experimental results show that our proposed mechanism outperforms the compared TOBA and BSAS mechanisms. Future work will consider mobile sensors and further optimize the surveillance quality of barrier coverage.

## Figures and Tables

**Table 1:** Comparisons between the proposed JSQE with the existing studies. ✗, Not have this property. ✓, Have this property.

Studies	Distributed	ESM Model	Monitoring Quality	Goal of Minimum Numbers of Sensors
[13]	✗	✗	✓	✗
[14]	✗	✗	✓	✗
[15]	✓	✗	✗	✗
[16]	✓	✗	✗	✓
[17]	✗	✓	✓	✗
[22]	✓	✓	✗	✗
JSQE	✓	✓	✓	✓

**Table 2:** Simulation parameters.

Parameter	Description
Monitoring area	400 m × 40 m
Number of sensor nodes	400–800
Sensing range	10 m
Communication range	20 m
Required monitoring quality	0.3, 0.5, 0.7
Working energy cost	0.05 J/s
Deployment	Randomly

## Data Availability

Not applicable.
